# Molecular and virulence characteristics of carbapenem-resistant *Acinetobacter baumannii* isolates: a prospective cohort study

**DOI:** 10.1038/s41598-023-46985-1

**Published:** 2023-11-09

**Authors:** Seung Min Park, Jin Woong Suh, Yong Kuk Ju, Jeong Yeon Kim, Sun Bean Kim, Jang Wook Sohn, Young Kyung Yoon

**Affiliations:** 1 Institute of Emerging Infectious Diseases, Korea University, Seoul, Republic of Korea; 2 Division of Infectious Diseases, Department of Internal Medicine, Korea University Anam Hospital, Korea University College of Medicine, 73 Inchon-ro, Seongbuk-gu, Seoul, 02841 Republic of Korea

**Keywords:** Microbiology, Medical research

## Abstract

This study aimed to characterize the molecular features and virulence profiles of carbapenem-resistant *Acinetobacter baumannii* (CRAB) isolates. Clinical CRAB isolates were obtained from blood cultures of adult patients with CRAB bacteremia, collected between July 2015 and July 2021 at a Korean hospital. Real-time polymerase chain reaction was used to detect 13 virulence genes, genotyping was conducted via multilocus sequence typing (MLST), and a *Tenebrio molitor* infection model was selected for survival analysis. Herein, 170 patients, from whom CRAB isolates were collected, showed the in-hospital mortality rate of 57.6%. All 170 clinical CRAB isolates harbored *bla*_OXA-23_ and *bla*_OXA-51_. MLST genotyping identified 11 CRAB sequence types (STs), of which ST191 was predominant (25.7%). Virulence genes were distributed as follows: *basD*, 58.9%; *espA*, 15.9%; *bap*, 92.4%; and *ompA*, 77.1%. In the *T. molitor* model, ST195 showed a significantly higher mortality rate (73.3% vs. 66.7%, p = 0.015) than the other groups. Our findings provide insights into the microbiological features of CRAB blood isolates associated with high mortality. We suggest a potential framework for using a *T. molitor* infection model to characterize CRAB virulence. Further research is warranted to elucidate the mechanisms by which virulence improves clinical outcomes.

## Introduction

*Acinetobacter baumannii* is a significant nosocomial pathogen with attributable mortality rates ranging from 8.4 to 36.5%^1^. Bacteremia and ventilator-associated pneumonia are major nosocomial infections caused by this pathogen. Owing to its multidrug resistance and virulence, *A. baumannii* is associated with unfavorable treatment outcomes. Carbapenem-resistant *A. baumannii* (CRAB) is ranked as a high-priority antibiotic-resistant pathogen and is designated as an urgent threat by the Centers for Disease Control and Prevention in the United States^2^. According to the Korean Antimicrobial Resistance Monitoring System and Korean Nosocomial Infections Surveillance System data, the resistance rate of *A. baumannii* to imipenem in the Republic of Korea (ROK) will increase to 85% in 2015 and 93.5% in 2022, thereby representing a major public health threat^3,4^.

Identifying the molecular features and mechanisms of virulence is crucial for containing transmission and reducing mortality associated with an increasing number of CRAB infections. In the ROK, a drastic increase in *Acinetobacter* isolates with *bla*_OXA-23_ genes has been observed since the mid-2000s^5^. Moreover, ST191 *A. baumannii* harboring *bla*_OXA-23_ has been held responsible for the high rate of carbapenem resistance recorded in the ROK between 2009 and 2012^6,7^. In addition to the complex mechanism of antibiotic resistance, CRAB carries several virulence genes that may result in clinical deterioration in patients with other co-morbidities; though, little is known about the distribution and clinical implications of the virulence genes in this bacterium. Previous studies suggested that several virulence genes are prevalent in *A. baumannii* isolates and may be associated with antibiotic resistance^8–11^. Recent evidence suggests that a hypervirulent strain of CRAB has evolved (theory of evolution), and is associated with high mortality and clonal transmission within hospitals^12–14^. However, few studies have focused on the distribution of virulence genes via multilocus sequence typing (MLST) of CRAB blood isolates. The aim of this study was to investigate the molecular characteristics of CRAB blood isolates and evaluate the survival rates of *Tenebrio molitor* that had been infected with CRAB strains exhibiting different molecular features.

## Methods

### Hospital setting and study design

This prospective cohort study was performed in a 1048-bed, university-affiliated hospital in ROK. Clinical CRAB isolates were obtained from blood cultures of adult patients (≥ 18 years of age) diagnosed with CRAB bacteremia between July 2015 and July 2021. Patients with polymicrobial bacteremia were excluded from the study. Patients had multiple episodes of CRAB bacteremia, and only the first episode was included. Clinical information associated with CRAB bacteremia was collected from the electronic medical records of each patient, including age, sex, source of CRAB bacteremia, and mortality. This study was approved by the Institutional Review Board of the Korea University Anam Hospital [No. 2022AN0292], and the requirement for written informed consent was waived. This study was conducted in accordance with the ethical guidelines and regulations outlined in the Declaration of Helsinki.

### Bacterial strains and antibiotic susceptibility test

All isolates were collected on day 1 of bacteremia. Non-repetitive CRAB isolates were identified using a MicroScan WalkAway-96 Plus system (Beckman Coulter, Inc., Fullerton, CA, USA) using a routine laboratory diagnostic process. Two reference bacterial strains, namely *Escherichia coli* ATCC® 25922 and *Pseudomonas aeruginosa* ATCC® 27853, were used for the internal quality control of the antibiotic susceptibility test. Minimal inhibitory concentrations (MICs) were determined using the agar dilution method and the breakpoints of the antibiotic susceptibility tests were interpreted according to the 2017 Clinical Laboratory Standards Institute guidelines^15^. Carbapenem resistance was defined as resistance to imipenem, at MICs of ≥ 8 μg/mL^15^.

### Virulence and antimicrobial resistant genes

Multiplex real-time polymerase chain reaction (PCR) assays were performed to detect virulence and antimicrobial resistance genes in CRAB isolates^16–18^. Thirteen virulence genes (b*asD, espA, ompA, bap, bfmR, pbpG, fhaB, cpaA, ata, recA, lipA, abeD,* and *chop*) and two carbapenemase genes (*bla*_OXA-23_ and *bla*_OXA-51_) were detected using specific primers and positive controls, which were previously confirmed as positive by sequencing (Supplementary Table S1). Virulence genes were associated with siderophore synthesis (*basD*), bacterial adhesion (*espA*, *bap*, *ata*, *chop*), biofilm formation (*ompA*, *pbpG*), glycoconjugates (*bfmR*), host cell death (*fhaB*, *abeD*), toxin production (*cpaA*, *lipA*), and stress response (*recA*)^19–21^. The PCR cycling conditions were as follows: denaturation at 95 ℃ for 5 min, annealing between 50 ℃ and 61 ℃ for 30–45 s, and extension at 72 ℃ for 1 min (for 34 cycles). The presence of amplified PCR products was determined by electrophoretic separation on 2% agarose gels and visualized using a transilluminator.

### Genotypes according to MLST

For MLST, seven housekeeping genes (*gltA, gyrB, gdhB, recA, cpn60, gpi,* and *rpoD*) of A. baumannii were amplified and sequenced for *A. baumannii*, as described previously^22^, using specific primers (Supplementary Table S2). MLST was performed using the Oxford scheme, and the sequence types (STs) were assigned using tools available in the *A. baumannii* MLST database (https://pubmlst.org/organisms/acinetobacter-baumannii).

### Virulence assay

A virulence assay was performed using *T. molitor* larvae (mealworms) as described previously^23^. Larvae were maintained in a plastic box containing wheat bran. CRAB isolates were placed in a brain–heart infusion broth and incubated overnight at 37 °C. Next, subcultures were grown on blood agar plates that were incubated for 24 h at 37 °C. The cultured CRAB were then suspended in insect saline (130 mM NaCl, 5 mM KCl, and 1 mM CaCl_2_) at a concentration of 10 CFU/mL. Using an insulin syringe, 10 µL of the CRAB suspension (at 10^6^ CFU/µl) was injected into a *T. molitor* larva; in the control group, 10 µL of sterilized insect saline solution was injected. However, a positive control group has not been established. Post injection, all larvae were raised in Petri dishes for 72 h at 25 °C. Survival rates were calculated according to melanization (using three sets of five larvae per strain).

### Statistical analyses

Categorical variables were summarized as frequencies and analyzed using Pearson’s chi-square or Fisher’s exact test. Continuous variables were expressed as medians with interquartile ranges and analyzed using the Student’s *t* test. The difference in the mortality rates of *T. molitor* larvae infected with CRAB isolates containing different STs was evaluated using a one-way analysis of variance. Statistical significance was set at p < 0.05. SPSS v23.0 for Windows software (SPSS Inc., Chicago, IL., USA) was used to conduct statistical analyses.

## Results

### Patients with CRAB bacteremia

During the study period, 170 patients, from whom CRAB isolates were collected, were enrolled. The mean age of the patients was 66 years (inter quartile range, 56–77 years), and 60.0% were male. The most common source of CRAB bacteremia was catheter-related bloodstream infections (50.6%), followed by primary bloodstream infections (23.5%), intra-abdominal infections (16.5%), pneumonia (8.2%), and skin and soft tissue infections (0.6%). Seventy-three patients (43.5%) experienced septic shock. The in-hospital and 30-day mortality rates were 57.6% and 21.2%, respectively.

### Antimicrobial susceptibility of CRAB isolates

A total of 170 CRAB blood isolates were collected; all were resistant to three or more antimicrobial agents and were therefore considered multidrug-resistant. Antibiotic resistance patterns of the isolates are listed in Table 1. The highest resistance rates were observed for ciprofloxacin and imipenem (100%), followed by those for piperacillin (99.4%), cefepime (99.4%), ampicillin/sulbactam (97.6%), gentamicin (90%), and minocycline (10%). *bla*_OXA-23_ and *bla*_OXA-51_ were identified in all strains (Table 2).

**Table 1:** MIC_50_ and MIC_90_ values and antimicrobial susceptibility (%) of CRAB isolates.

Antibiotics	MIC (mg/L)			Percentage of susceptible isolates (%)
	MIC_50_	MIC_90_	Range	
Imipenem	≥ 16	≥ 16	> 8 to ≥ 16	0
Ampicillin/sulbactam	> 16	≥ 32	4 to ≥ 32	2.4
Minocycline	≤ 1	8	≤ 0.5 to ≥ 16	90.0
Ciprofloxacin	≥ 4	≥ 4	≥ 2 to ≥ 4	0
Piperacillin	≥ 128	≥ 128	≥ 64 to ≥ 128	0.6
Cefepime	≥ 64	≥ 64	≥ 8 to ≥ 64	0.6
Gentamicin	> 16	≥ 16	≥ 1 to ≥ 16	10.0

*MIC* minimum inhibitory concentration.

**Table 2:** Distribution of virulence and antimicrobial resistant genes according to CRAB isolate sequence types.

N (%)	ST191 (n = 49)	ST195 (n = 28)	ST208 (n = 9)	ST357 (n = 1)	ST369 (n = 7)	ST451 (n = 27)	ST469 (n = 2)	ST491 (n = 4)	ST784 (n = 23)	ST1599 (n = 9)	ST1653 (n = 1)	NT (n = 10)
Virulence gene, n (%)												
*basD* (100)	30 (61.2)	26 (92.9)	2 (22.2)	1 (100)	6 (85.7)	12 (44.4)	2 (100)	3 (75.0)	9 (39.1)	2 (22.2)	1 (100)	6 (60.0)
*espA* (27)	7 (14.3)	7 (25.0)	0	0	7 (100)	2 (7.41)	0	0	1 (4.35)	2 (22.2)	0	1 (100)
*ompA* (131)	40 (81.6)	28 (100)	2 (22.2)	1 (100)	6 (85.7)	18 (66.7)	2 (100)	4 (100)	11 (47.8)	9 (100)	1 (100)	9 (90.0)
*bap* (157)	47 (95.9)	26 (92.9)	9 (100)	1 (100)	7 (100)	27 (100)	2 (100)	1 (25.0)	23 (100)	9 (100)	1 (100)	4 (40.0)
*bfmR* (158)	45 (91.8)	27 (96.4)	8 (88.9)	1 (100)	7 (100)	27 (100)	2 (100)	4 (100)	17 (13.9)	9 (100)	1 (100)	10 (100)
*pbpG* (159)	44 (89.8)	27 (96.4)	9 (100)	1 (100)	7 (100)	25 (92.6)	2 (100)	4 (100)	21 (91.3)	9 (100)	1 (100)	9 (90.0)
*fhaB* (120)	48 (98.0)	1 (3.6)	9 (100)	1 (100)	7 (100)	27 (100)	2 (100)	0	13 (56.5)	9 (100)	0	3 (30.0)
*cpaA* (1)	1 (2.04)	0	0	0	0	0	0	0	0	0	0	0
*ata* (147)	48 (98.0)	26 (92.9)	9 (100)	1 (100)	2 (28.6)	26 (96.3)	2 (100)	0	22 (95.7)	8 (88.9)	1 (100)	2 (20.0)
*recA* (170)	49 (100)	28 (100)	9 (100)	1 (100)	7 (100)	27 (100)	2 (100)	4 (100)	23 (100)	9 (100)	1 (100)	10 (100)
*lipA* (169)	49 (100)	27 (96.4)	9 (100)	1 (100)	7 (100)	27 (100)	2 (100)	4 (100)	23 (100)	9 (100)	1 (100)	10 (100)
*abeD* (169)	49 (100)	27 (96.4)	9 (100)	1 (100)	7 (100)	27 (100)	2 (100)	4 (100)	23 (100)	9 (100)	1 (100)	10 (100)
*chop* (12)	1 (2.0)	2 (7.1)	0	0	1 (14.3)	1 (3.7)	0	3 (75.0)	0	0	0	4 (40.0)
Antimicrobial resistant gene, n (%)												
*bla*_OXA-23_ (170)	49 (100)	28 (100)	9 (100)	1 (100)	7 (100)	27 (100)	2 (100)	4 (100)	23 (100)	9 (100)	1 (100)	10 (100)
*bla*_OXA-51_ (170)	49 (100)	28 (100)	9 (100)	1 (100)	7 (100)	27 (100)	2 (100)	4 (100)	23 (100)	9 (100)	1 (100)	10 (100)

*ST* sequence type.

### MLST analysis of CRAB isolates

**Figure 1:**
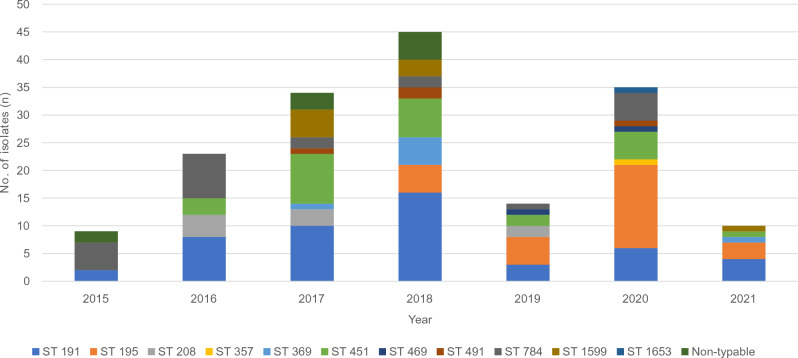
The distribution of multilocus sequence types (STs) among carbapenem-resistant *A. baumannii* blood isolates, according to the year of isolation.

MLST analysis revealed 11 different STs in 160 (94.1%) CRAB isolates: ST191, ST195, ST208, ST357, ST369, ST451, ST469, ST491, ST784, ST1599, and ST1653 (Supplementary Table S3). Of these, ST191 was the most abundant, occurring in 49 (28.8%) isolates. ST191 was mostly isolated between 2015 and 2018, whereas ST195 was mainly found in samples collected between 2019 and 2021. ST451 (n = 27) was detected throughout the study. ST491 (n = 4), ST1599 (n = 9), ST469 (n = 2), and ST357 (n = 1) were detected after 2017 (Fig. 1).

### Distribution of virulence factors in CRAB isolates

The distribution of virulence genes was as follows: 58.9% *basD* (siderophore synthesis); 15.9% *espA*, 92.4% *bap*, 86.5% *ata*, and 7.1% *chop* (bacterial adhesion); 77.1% *ompA* and 93.5% *pbpG* (biofilm formation); 92.9% *bfmR* (glycoconjugates); 70.6% *fhaB* and 99.4% *abeD* (host cell death); 0.6% *cpaA* and 99.4% *lipA* (toxin production); and 100% *recA* (stress response) (Table 2). We found that one isolate carried 12 virulence genes, 11 isolates carried 11 virulence genes, 49 isolates carried 10 virulence genes, 52 isolates carried nine virulence genes, 37 isolates carried eight virulence genes, 14 isolates carried seven virulence genes, five isolates carried six virulence genes, and one isolate carried five virulence genes. Occasionally, the frequency of a virulence gene differed according to the ST (Table 2).

### Virulence assay

The overall mortality rate of CRAB-inoculated *T. molitor* was 70.4%. The mortality rates in our *T. molitor* model showed the following differences depending on the virulence genes carried by CRAB isolates: 71.9% (1079/1500) *basD* (siderophore synthesis); 72.6% (294/405) *espA*, 68.8% (1620/2355) *bap*, 68.1% (1502/2205) *ata*, and 71.7% (129/180) *chop* (bacterial adhesion); 69.9% (1374/1965) *ompA* and 68.1% (1625/2385) *pbpG* (biofilm formation); 68.3% (1619/2370) *bfmR* (glycoconjugates); 67.9% (1222/1800) *fhaB* and 68.5% (1737/2535) *abeD* (host cell death); 80.0% (12/15) *cpaA* and 68.5% (1737/2535) *lipA* (toxin production); and 68.5% (1747/2550) *recA* (stress response). The mortality rates of *T. molitor* larvae infected with CRAB isolates were statistically significantly different among the different STs (Table 3): ST1599 vs. ST191 (p = 0.004), ST1599 vs. ST195 (p = 0.002), ST1599 vs. ST208 (p = 0.042), and ST1599 vs. ST369 (p = 0.01).

**Table 3:** Comparison of mortality rates in our *T. molitor* model according to different CRAB isolate sequence types.

No.	ST191	ST195	ST208	ST357	ST369	ST451	ST469	ST491	ST784	ST1599	ST1653	Non-typable
	49	28	9	1	7	27	2	4	23	9	1	10
Mortality rates (%)	71.4	73.3	72.6	73.3	77.1	61.7	76.7	76.7	65.2	51.8	80	65.3
ST191												
ST195	0.495											
ST208	0.795	0.852										
ST357	0.883	0.998	0.952									
ST369	0.265	0.406	0.460	0.795								
ST451	**0.003**	**0.001**	**0.043**	0.429	**0.014**							
ST469	0.574	0.679	0.695	0.926	0.969	0.175						
ST491	0.415	0.531	0.506	0.685	0.947	**0.049**	1.000					
ST784	0.078	**0.044**	0.219	0.630	0.088	0.420	0.360	0.182				
ST1599	** < 0.001**	** < 0.001**	**0.002**	0.163	**0.002**	0.074	0.060	**0.005**	**0.036**			
ST1653	0.499	0.522	0.532	–	0.847	0.216	0.926	0.685	0.381	0.078		
Non-typable	0.207	0.238	0.334	0.703	0.183	0.539	0.477	0.284	0.987	0.098	0.488	

Significant values are in bold.

### Differences in microbiological characteristics of CRAB among major STs

CRAB isolates with ST191 displayed a significantly lower rate of resistance to minocycline than the non-ST191 group (2.0% vs. 13.3%, p = 0.027), whereas ST451 isolates showed a higher rate of resistance to minocycline than the non-ST451 group (33.3% vs. 5.6%, p < 0.001). The ST195 group showed a lower tigecycline resistance rate than the non-ST195 group (4.2% vs. 22.1%, p = 0.042). Additionally, the ST191 group showed a higher prevalence than the non-ST191 group in virulence genes such as *fhaB* (host cell death; 98% vs. 59.5%, p < 0.001) and *ata* (bacterial adhesion; 98.0% vs. 81.8%, p = 0.005). In contrast, the ST195 group was more predominant than the non-ST195 group in terms of *basD* (siderophore synthesis: 92.9% vs. 52.1%, p < 0.001) and *ompA* (biofilm formation: 100% vs. 72.5%, p = 0.002) genes. Notably, the ST195 group showed a significantly higher *T. molitor* mortality rate than the non-ST195 group (73.3 vs. 66.7%, p = 0.015), whereas the mortality rate was lower in the ST451 group than in the non-ST451 group (60.0% vs. 73.3%, p = 0.007) (Table 4).

**Table 4:** Comparison of microbiological characteristics according to the main CRAB blood isolate sequence types.

	ST191 (n = 49)	Non-ST191 (n = 121)	p value	ST195 (n = 28)	Non-ST195 (n = 142)	p value	ST451 (n = 27)	Non-ST451 (n = 143)	p value
Antimicrobial resistance, n (%)									
Imipenem	49 (100)	121 (100)	–	28 (100)	142 (100)		27 (100)	143 (100)	–
Ampicillin/sulbactam	48 (98.0)	118 (97.5)	0.864	28 (100)	138 (97.2)	0.369	25 (92.6)	141 (98.6)	0.059
Minocycline	1 (2.0)	16 (13.3)	0.027	1 (3.6)	16 (11.3)	0.211	9 (33.3)	8 (5.6)	< 0.001
Virulence genes, n (%)									
*basD*	30 (61.2)	70 (57.9)	0.686	26 (92.9)	74 (52.1)	< 0.001	12 (44.4)	88 (61.5)	0.098
*espA*	7 (14.3)	20 (16.5)	0.717	7 (25.0)	20 (14.1)	0.149	2 (7.4)	25 (17.5)	0.189
*ompA*	40 (81.6)	91 (75.2)	0.367	28 (100)	103 (72.5)	0.002	18 (66.7)	113 (79.0)	0.161
*bap*	47 (95.9)	110 (90.9)	0.266	26 (92.9)	131 (92.3)	0.913	27 (100)	130 (90.9)	0.103
*bfmR*	45 (91.8)	113 (93.4)	0.721	27 (96.4)	131 (92.3)	0.431	27 (100)	131 (91.6)	0.118
*pbpG*	44 (89.8)	115 (95.0)	0.208	27 (96.4)	132 (93.0)	0.495	25 (92.6)	134 (93.7)	0.829
*fhaB*	48 (98.0)	72 (59.5)	< 0.001	1 (3.6)	119 (83.8)	< 0.001	27 (100)	93 (65.0)	< 0.001
*cpaA*	1 (2.0)	0	0.115	0	1 (0.7)	0.656	0	1 (0.7)	0.663
*ata*	48 (98.0)	99 (81.8)	0.005	26 (92.9)	121 (85.2)	0.280	26 (96.3)	121 (84.6)	0.104
*recA*	49 (100)	121 (100)	–	28 (100)	142 (100)	–	27 (100)	143 (100)	–
*lipA*	49 (100)	120 (99.2)	0.523	27 (96.4)	142 (100)	0.024	27 (100)	142 (99.3)	0.663
*abeD*	49 (100)	120 (99.2)	0.523	27 (96.4)	142 (100)	0.024	27 (100)	142 (99.3)	0.663
*chop*	1 (2.0)	11 (9.1)	0.104	2 (7.1)	10 (7.0)	0.985	1 (3.7)	11 (7.7)	0.458
*T. molitor* mortality rates % (IQR)	73.3 (60–80)	66.7 (60–80)	0.092	73.3 (66.7–80)	66.7 (60–80)	0.015	60.0 (46.7–73.3)	73.3 (60–80)	0.007

*IQR* interquartile range.

## Discussion

We demonstrated an evolutionary change in CRAB from blood isolates collected from a hospital, as evidenced by the emergence of new bacterial STs. During the study period, ST191, harboring the *bla*_OXA-23_ carbapenemase gene, was the most prevalent genotype, and a marked increase in genomic variation was observed after 2019. To the best of our knowledge, this study is the first to evaluate the virulence of CRAB isolates and the associated mortality in a *T. molitor* larval infection model.

CRAB isolates displayed an extensive drug-resistant phenotype, being highly resistant to all clinically available antimicrobial agents, which is consistent with previous findings in the ROK^9,24^. Our results also revealed that all CRAB isolates harbored *bla*_OXA-23_, which previous studies have identified as the most prevalent carbapenemase gene carried by *A. baumannii* in Asian countries^25–28^. Our findings suggest that the genetic evolution enabling *bla*_OXA-23_ expression in CRAB contributes to this bacterium, establishing it as a highly successful nosocomial pathogen in clinical settings.

Furthermore, ST191 was the predominant CRAB isolate until 2018, whereafter ST195 became more prevalent. In 2018, ST195 was the most prevalent ST in China^29^. The same shift in the predominant ST from ST191 to ST195 was also reported in Hong Kong following the emergence of CRAB ST195 harboring the *bla*_OXA-23_ gene^30^. Our study showed that the ST195 harboring the *bla*_OXA-23_ and *bla*_OXA-51_ genes was associated with higher mortality in a *T. molitor* infection model, compared to those of the non-ST195. The shift in the predominant sequence type of CRAB strains from ST191 to ST195 may be an adaptation through the acquisition of higher virulence. In contrast, our study demonstrated that ST451 first emerged in 2016 and was steadily discovered during the study period. Similarly, a previous study in the ROK reported that ST451 was the most prevalent ST between 2016 and 2018 in the context of clonal evolution related to antimicrobial resistance^9^. Indeed, all the prevalent STs recorded (ST191, ST195, and ST451—are well-known multidrug-resistant clones, causing a decrease in the likelihood of appropriate antibiotic selection^11^. There appears to be a clonal spread of several epidemic lineages that predominate over the rest and play a pivotal role in nosocomial transmission.

Several virulence factors may influence disease progression to critical illness and are related to functions, such as transmission, binding to host structures, cellular damage, and invasion. Consistent with previous studies, we found that stress response-associated *recA* was present in all CRAB isolates^10^. The remaining virulence genes showed differential distributions according to ST. A correlation between virulence genes and multidrug-resistant phenotypic differences in CRAB isolates has been previously suggested^9–11^. Selection pressure may drive the formation of virulence factors and the acquisition of multidrug resistance during the process of adaptation, facilitating the survival of these isolates in a clinical setting.

Previous reports suggested a positive relationship between the expression of virulence genes associated with biofilm formation and antibiotic resistance in *A. baumannii* isolates^31–33^. Biofilm formation is responsible for various types of medical device-related infections. Notably, CRAB strains, as biofilm-formers, are also advantageous for survival and dissemination in the hospital environment and the acquisition of antimicrobial resistance^33^. Although our study did not confirm the biofilm-forming capacity of the CRAB isolates, we assessed the frequency of biofilm-associated gene occurrence in CRAB harboring *bla*_OXA-23_, recording 15.9, 92.4, 86.5, 7.1, 77.1, and 93.5% for *espA*, *bap*, *ata*, *chop, ompA*, and *pbpG*, respectively. In particular, ompA and bap are essential for bacterial adhesion to human epithelial cells, development of biofilms, and antimicrobial resistance^34,35^. The virulence genes identified in our study may lead to treatment challenges owing to their high pathogenicity and antimicrobial resistance^36^. In Iran and Korea, *A. baumannii* isolates harboring *ompA* (81% and 69%) and *bap* (92% and 100%), respectively, have been similarly identified^32,37–39^. Surveillance and control measures against CRAB isolates containing biofilm-associated genes are essential to prevent their emergence and transmission.

Furthermore, we evaluated the virulence of the CRAB blood isolates using *T. molitor* larvae as an infection model. Mealworms are widely used to assess the pathogenic virulence of bacteria such as *Listeria monocytogenes, Staphylococcus aureus,* and *Aeromonas hydrophila*^40–42^. *Galleria mellonella* is another reliable model for the evaluation of CRAB strain pathogenicity^43^. Although none of the insect species can replace mammalian models, *T. molitor* larvae offer an attractive alternative for investigating host–pathogen interactions involving CRAB isolates because of their cost-effectiveness and ease of handling for housing and breeding^44^. In addition, its short lifecycle allows for large-scale experiments over a short period. In addition to sharing all of the merits already listed for *G. mellonella*, *T. molitor* larvae are larger.

Interestingly, our study showed that the mortality rates of *T. molitor* larvae infected with CRAB isolates differed according to the ST. The relative rarity of some STs, resulting in a correspondingly small sample size of related blood isolates, prevented us from drawing definite conclusions. Nevertheless, ST195 larvae exhibited a significantly higher mortality rate than the non-ST195 group, whereas ST451 mealworms exhibited a lower mortality rate than the non-ST451 group. ST191 also displayed a trend of a higher mortality rate among larvae than the non-ST191 group, although this difference was not statistically significant. A previous study reported that patients infected with ST191, ST195 or ST208 may develop severe infections accompanied by organ damage^45^. Furthermore, ST191 and ST195 strains are considered highly virulent in serum complement killing assays and *G. mellonella* models^45,46^. In a clinical study with similar results, Cox regression multivariate analysis found that ST191 was a risk factor associated with 30-day mortality in patients with *A. baumannii* bloodstream infections, whereas ST451 served as a protective factor^11^. The distribution of STs varies according to geographical location^14,47^. In Taiwan, ST218 harboring *bla*_OXA-72_ gene (which was not detected in our study) was also reported as a hypervirulent strain, raising the possibility of intra-hospital transmission and mortality^14^.

Our study demonstrated that ST191 isolates exhibited a significantly lower resistance rate to minocycline than non-ST191 isolates, whereas ST451 showed the opposite. Considering that the most dominant ST is shifting from ST191 to ST451, it is evident that CRAB isolates are becoming more multidrug-resistant and virulent. Consequently, delays in appropriate antibiotic administration and rapid disease progression may have synergistic effects, contributing to treatment failure. Antibiotic susceptibility, genetic type, and virulence genes of CRAB blood isolates that constitute the microbiological characteristics may be related to each other.

Our study has some limitations. First, all CRAB blood isolates were collected from a single hospital in the ROK, and generalizing the results to other regions or healthcare facilities may be problematic. Further studies with larger sample sizes and in different clinical settings are necessary to fully understand the microbiological characteristics of CRAB. Second, we analyzed only a limited number of virulence types and antibiotic resistance genes, disregarding other factors that could potentially contribute to CRAB infection pathogenicity. In addition, the presence of virulence and antibiotic resistance genes may not necessarily indicate their expression. However, clear evidence is needed to confirm whether their expression is related to pathogenicity. Finally, our virulence assay relied on an insect model, which may not accurately reflect the virulence of human CRAB isolates. Compared to non-ST191, the relatively high mortality rate of ST191 identified in *T. molitor* infection models should be verified in patients with CRAB bacteremia. In future, various clinical variables and microbiological characteristics should be simultaneously considered as confounding variables to identify significant risk factors related to treatment outcomes.

## Conclusion

Our findings showed that ST191 and ST195, which are associated with high antimicrobial resistance and virulence in the ROK, are prevalent in clinical settings. Insights into the dynamic changes in antibiotic resistance mechanisms and virulence factors among CRAB isolates are necessary for the effective control and treatment of CRAB infections. We propose the use of the *T. molitor* infection model as an alternative approach for evaluating the pathogenicity of CRAB isolates. Research with larger sample sizes obtained from a variety of settings is essential to further explore the mechanism of virulence of CRAB isolates to reduce the mortality associated with CRAB bacteremia.

### Supplementary Information

Supplementary Tables.

## Data Availability

The datasets generated in the current study are available in the NCBI GenBank database under accession numbers OR498912–OR499081, [https://www.ncbi.nlm.nih.gov/nucleotide/]. Further information is provided by the corresponding author upon request.
