# Supplementary material for: Molecular and virulence characteristics of carbapenem-resistant Acinetobacter baumannii isolates: a prospective cohort study
Source: Sci Rep. 2023 Nov 9;13:19536. doi: 10.1038/s41598-023-46985-1 (PMC10636183; doi:10.1038/s41598-023-46985-1)
Supplement: Supplementary file 1 — Supplementary Tables. [file 41598_2023_46985_MOESM1_ESM.docx]

**Supplementary Table S1.** The primers used for identifying virulence and antimicrobial resistance genes of CRAB isolates

|  |  | **Sequence (5´ 🡪 3´)** | **Size (bp)** |
| --- | --- | --- | --- |
| **Virulence genes** | | | |
| *ompA* | F | GCTGGTGTTGGTGCTTTCTG | 490 |
|  | R | TCGGTTGATCCCAAGCGAAA |  |
| *bap* | F | TGAAAGTGGCTGCCAGTGAT | 223 |
|  | R | TCTGCGTCAGCGTCACTATC |  |
| *espA* | F | CAGCTTTAGGTCTTGCGGGA | 392 |
|  | R | TGCTAAACCGAAGTCGCCAA |  |
| *pbpG* | F | TGGATGCGCAAACAGGTGAA | 467 |
|  | R | GGTCGGTGTGGTTGAGAACT |  |
| *basD* | F | TGCTGTTCGTTCTTTTGGCG | 517 |
|  | R | GTTGAGTTTGAGCGCCGATG |  |
| *bfmR* | F | ACCGATGGTAACCGTGCAAT | 194 |
|  | R | TCTGCACCCATTTCCAGACC |  |
| *ompA* | F | GCTGGTGTTGGTGCTTTCTG | 490 |
|  | R | TCGGTTGATCCCAAGCGAAA |  |
| *bap* | F | TGAAAGTGGCTGCCAGTGAT | 223 |
|  | R | TCTGCGTCAGCGTCACTATC |  |
| *espA* | F | CAGCTTTAGGTCTTGCGGGA | 392 |
|  | R | TGCTAAACCGAAGTCGCCAA |  |
| *cpaA* | F | CTGCTTTAGGAAAATGGG | 668 (599-1267) |
|  | R | CGCCTTCAATCATTCTAAG |  |
| *recA* | F | CACGCCCTAGACCCTCAATA | 136 |
|  | R | CGATTAAATCAATTGCGCCT |  |
| *fhaB* | F | ACTCAGCAGGTACAGTCGTTCC | 1435 |
|  | R | GCCTTGTCAGCATTGTTCCAGA |  |
| *ata* | F | ATCCAACAACCGAAGCGAATCA | 398 |
|  | R | ACCACCTACAGCAGCAGCAT |  |
| *lipA* | F | TGCHMARACMAARTATCC | 770 |
|  | R | AYYTGATTSACTTCATC |  |
| *abeD* | F | TTGGCTTGCCAAATGACGTG | 247 |
|  | R | TCTTGACGACTAACCGCACC |  |
| *choP* | F | GCAGCGTGACTCCATTCGTTA | 344 |
|  | R | TGTAGCCCAAAGAAACCGTATGAT |  |
| **Antimicrobial resistance genes** | | | |
| *bla*_OXA-23_ | F | GATCGGATTGGAGAACCAGA | 501 |
|  | R | ATTTCTGACCGCATTTCCAT |  |
| *bla*_OXA-51_ | F | TAATGCTTTGATCGGCCTTG | 353 |
|  | R | TGGATTGCACTTCATCTTGG |  |

CRAB, carbapenem-resistant *Acinetobacter baumannii*

**Supplementary Table S2.** The primers used for identifying multilocus sequence typing of CRAB isolates

|  |  | **Sequence (5´ 🡪 3´)** | **Size (bp)** |
| --- | --- | --- | --- |
| **Virulence genes** | | | |
| *gltA* | F | AATTTACAGTGGCACATTAGGTCCC | 722 |
|  | R | GCAGAGATACCAGCAGAGATACACG |  |
| *gyrB* | F | TGAAGGCGGCTTATCTGAGT | 594 |
|  | R | GCTGGGTCTTTTTCCTGACA |  |
| *gdhB* | F | GCTACTTTTATGCAACAGAGCC | 774 |
|  | R | GTTGAGTTGGCGTATGTTGTGC |  |
| *recA* | F | CCTGAATCTTCYGGTAAAAC | 425 |
|  | R | GTTTCTGGGCTGCCAAACATTAC |  |
| *cpn60* | F | GGTGCTCAACTTGTTCGTGA | 640 |
|  | R | CACCGAAACCAGGAGCTTTA |  |
| *gpi* | F | GAAATTTCCGGAGCTCACAA | 456 |
|  | R | TCAGGAGCAATACCCCACTC |  |
| *rpoD* | F | ACCCGTGAAGGTGAAATCAG | 672 |
|  | R | TTCAGCTGGAGCTTTAGCAAT |  |

CRAB, carbapenem-resistant *Acinetobacter baumannii*

**Supplementary Table S3.** The allelic profiles of seven housekeeping genes for multilocus sequence typing of CRAB isolates.

|  | GenBank accession number | Source | Date | *gltA* | *gyrB* | *gdhB* | *recA* | *cpn60* | *gpi* | *rpoD* | ST |
| --- | --- | --- | --- | --- | --- | --- | --- | --- | --- | --- | --- |
| 1 | OR498912 | Blood | 2015-09-17 | 1 | 3 | 3 | 77 | 127 | 94 | 3 | NT |
| 2 | OR498913 | Blood | 2015-10-08 | 1 | 3 | 3 | 2 | 2 | 94 | 3 | 191 |
| 3 | OR498914 | Blood | 2015-10-13 | 1 | 3 | 3 | 2 | 2 | 94 | 3 | 191 |
| 4 | OR498915 | Blood | 2015-12-09 | 21 | 12 | 2 | 28 | 23 | 306 | 4 | NT |
| 5 | OR498916 | Blood | 2015-12-28 | 1 | 3 | 3 | 2 | 2 | 107 | 3 | 784 |
| 6 | OR498917 | Blood | 2015-12-31 | 1 | 3 | 3 | 2 | 2 | 107 | 3 | 784 |
| 7 | OR498918 | Blood | 2016-01-13 | 1 | 3 | 3 | 2 | 2 | 107 | 3 | 784 |
| 8 | OR498919 | Blood | 2016-01-19 | 1 | 3 | 3 | 2 | 2 | 107 | 3 | 784 |
| 9 | OR498920 | Blood | 2016-01-23 | 1 | 3 | 3 | 2 | 2 | 107 | 3 | 784 |
| 10 | OR498921 | Blood | 2016-02-04 | 1 | 3 | 3 | 2 | 2 | 107 | 3 | 784 |
| 11 | OR498922 | Blood | 2016-03-03 | 1 | 3 | 3 | 2 | 2 | 94 | 3 | 191 |
| 12 | OR498923 | Blood | 2016-05-04 | 1 | 3 | 3 | 2 | 2 | 97 | 3 | 208 |
| 13 | OR498924 | Blood | 2016-06-22 | 1 | 3 | 3 | 2 | 2 | 94 | 3 | 191 |
| 14 | OR498925 | Blood | 2016-06-23 | 1 | 3 | 3 | 2 | 2 | 94 | 3 | 191 |
| 15 | OR498926 | Blood | 2016-07-05 | 1 | 3 | 3 | 2 | 2 | 94 | 3 | 191 |
| 16 | OR498927 | Blood | 2016-07-22 | 1 | 3 | 3 | 2 | 2 | 107 | 3 | 784 |
| 17 | OR498928 | Blood | 2016-08-05 | 1 | 3 | 3 | 2 | 2 | 107 | 3 | 784 |
| 18 | OR498929 | Blood | 2016-08-18 | 1 | 3 | 3 | 2 | 2 | 94 | 3 | 191 |
| 19 | OR498930 | Blood | 2016-09-05 | 1 | 3 | 3 | 2 | 2 | 107 | 3 | 784 |
| 20 | OR498931 | Blood | 2016-09-06 | 1 | 3 | 3 | 2 | 2 | 107 | 3 | 784 |
| 21 | OR498932 | Blood | 2016-09-12 | 1 | 3 | 3 | 2 | 2 | 94 | 3 | 191 |
| 22 | OR498933 | Blood | 2016-09-26 | 1 | 3 | 3 | 2 | 2 | 94 | 3 | 191 |
| 23 | OR498934 | Blood | 2016-10-05 | 1 | 3 | 3 | 2 | 2 | 97 | 3 | 208 |
| 24 | OR498935 | Blood | 2016-10-05 | 1 | 3 | 3 | 2 | 2 | 107 | 3 | 784 |
| 25 | OR498936 | Blood | 2016-10-06 | 1 | 3 | 3 | 2 | 2 | 97 | 3 | 208 |
| 26 | OR498937 | Blood | 2016-10-21 | 1 | 3 | 3 | 2 | 2 | 142 | 3 | 451 |
| 27 | OR498938 | Blood | 2016-12-07 | 1 | 3 | 3 | 2 | 2 | 107 | 3 | 784 |
| 28 | OR498939 | Blood | 2016-12-22 | 1 | 3 | 3 | 2 | 2 | 94 | 3 | 191 |
| 29 | OR498940 | Blood | 2017-01-13 |  | 3 | 3 | 2 | 2 | 107 | 3 | 784 |
| 30 | OR498941 | Blood | 2017-01-17 | 1 | 3 | 3 | 2 | 2 | 97 | 3 | 208 |
| 31 | OR498942 | Blood | 2017-01-24 | 1 | 3 | 3 | 2 | 2 | 142 | 3 | 451 |
| 32 | OR498943 | Blood | 2017-02-28 | 1 | 3 | 3 | 2 | 2 | 142 | 3 | 451 |
| 33 | OR498944 | Blood | 2017-03-07 | 1 | 3 | 3 | 2 | 2 | 106 | 3 | 369 |
| 34 | OR498945 | Blood | 2017-03-17 | 1 | 3 | 3 | 2 | 2 | 107 | 3 | 784 |
| 35 | OR498946 | Blood | 2017-04-08 | 1 | 3 | 3 | 2 | 2 | 142 | 3 | 451 |
| 36 | OR498947 | Blood | 2017-05-10 | 1 | 3 | 3 | 2 | 2 | 142 | 3 | 451 |
| 37 | OR498948 | Blood | 2017-05-19 | 1 | 3 | 3 | 2 | 2 | 94 | 3 | 191 |
| 38 | OR498949 | Blood | 2017-05-20 | 1 | 54 | 62 | 31 | 4 | 78 | 45 | NT |
| 39 | OR498950 | Blood | 2017-05-20 | 1 | 3 | 3 | 2 | 2 | 97 | 3 | 208 |
| 40 | OR498951 | Blood | 2017-05-23 | 1 | 3 | 3 | 2 | 2 | 97 | 3 | 208 |
| 41 | OR498952 | Blood | 2017-06-05 | 1 | 3 | 3 | 2 | 2 | 142 | 3 | 451 |
| 42 | OR498953 | Blood | 2017-06-05 | 1 | 3 | 3 | 2 | 2 | 142 | 3 | 451 |
| 43 | OR498954 | Blood | 2017-07-06 | 1 | 3 | 3 | 2 | 2 | 142 | 3 | 451 |
| 44 | OR498955 | Blood | 2017-07-06 | 1 | 3 | 3 | 2 | 2 | 97 | 3 | 208 |
| 45 | OR498956 | Blood | 2017-07-19 | 1 | 3 | 3 | 2 | 2 | 94 | 3 | 191 |
| 46 | OR498957 | Blood | 2017-07-21 | 1 | 3 | 3 | 2 | 2 | 142 | 3 | 451 |
| 47 | OR498958 | Blood | 2017-07-22 | 1 | 3 | 3 | 2 | 2 | 94 | 3 | 191 |
| 48 | OR498959 | Blood | 2017-07-26 | 1 | 54 | 62 | 31 | 4 | 395 | 45 | NT |
| 49 | OR498960 | Blood | 2017-08-02 | 1 | 54 | 62 | 31 | 4 | 76 | 45 | NT |
| 50 | OR498961 | Blood | 2017-08-17 | 1 | 3 | 3 | 2 | 2 | 94 | 3 | 191 |
| 51 | OR498962 | Blood | 2017-08-22 | 1 | 3 | 3 | 2 | 2 | 94 | 3 | 191 |
| 52 | OR498963 | Blood | 2017-09-02 | 1 | 3 | 3 | 2 | 2 | 142 | 3 | 451 |
| 53 | OR498964 | Blood | 2017-09-07 | 1 | 3 | 3 | 2 | 2 | 142 | 3 | 451 |
| 54 | OR498965 | Blood | 2017-09-11 | 1 | 3 | 3 | 2 | 2 | 94 | 3 | 191 |
| 55 | OR498966 | Blood | 2017-09-14 | 1 | 3 | 3 | 2 | 2 | 94 | 3 | 191 |
| 56 | OR498967 | Blood | 2017-10-16 | 1 | 12 | 3 | 2 | 2 | 98 | 3 | 1599 |
| 57 | OR498968 | Blood | 2017-10-20 | 1 | 12 | 3 | 2 | 2 | 98 | 3 | 1599 |
| 58 | OR498969 | Blood | 2017-10-26 | 1 | 12 | 3 | 2 | 2 | 98 | 3 | 1599 |
| 59 | OR498970 | Blood | 2017-11-03 | 1 | 12 | 3 | 2 | 2 | 98 | 3 | 1599 |
| 60 | OR498971 | Blood | 2017-11-25 | 1 | 3 | 3 | 2 | 2 | 94 | 3 | 191 |
| 61 | OR498972 | Blood | 2017-12-02 | 10 | 53 | 4 | 11 | 4 | 98 | 5 | 491 |
| 62 | OR498973 | Blood | 2017-12-02 | 1 | 3 | 3 | 2 | 2 | 142 | 3 | 451 |
| 63 | OR498974 | Blood | 2017-12-19 | 1 | 3 | 3 | 2 | 2 | 107 | 3 | 784 |
| 64 | OR498975 | Blood | 2017-12-19 | 1 | 3 | 3 | 2 | 2 | 94 | 3 | 191 |
| 65 | OR498976 | Blood | 2018-01-23 | 1 | 3 | 3 | 2 | 2 | 94 | 3 | 191 |
| 66 | OR498977 | Blood | 2018-01-23 | 1 | 3 | 3 | 2 | 2 | 142 | 3 | 451 |
| 67 | OR498978 | Blood | 2018-09-25 | 1 | 3 | 3 | 2 | 2 | 94 | 3 | 191 |
| 68 | OR498979 | Blood | 2018-01-31 | 1 | 3 | 3 | 2 | 2 | 142 | 3 | 451 |
| 69 | OR498980 | Blood | 2018-12-10 | 1 | 3 | 3 | 2 | 2 | 96 | 3 | 191 |
| 70 | OR498981 | Blood | 2018-02-08 | 1 | 3 | 3 | 2 | 2 | 94 | 3 | 191 |
| 71 | OR498982 | Blood | 2018-12-15 | 1 | 3 | 3 | 2 | 2 | 96 | 3 | 195 |
| 72 | OR498983 | Blood | 2018-02-10 | 1 | 3 | 3 | 2 | 2 | 94 | 3 | 191 |
| 73 | OR498984 | Blood | 2018-02-15 | 1 | 3 | 3 | 2 | 2 | 94 | 3 | 191 |
| 74 | OR498985 | Blood | 2018-02-21 | 1 | 3 | 3 | 2 | 2 | 94 | 3 | 191 |
| 75 | OR498986 | Blood | 2018-02-22 | 1 | 3 | 3 | 2 | 2 | 142 | 3 | 451 |
| 76 | OR498987 | Blood | 2018-02-24 | 1 | 3 | 3 | 2 | 2 | 94 | 3 | 191 |
| 77 | OR498988 | Blood | 2018-02-24 | 1 | 12 | 3 | 2 | 2 | 98 | 3 | 1599 |
| 78 | OR498989 | Blood | 2018-02-26 | 1 | 12 | 3 | 2 | 2 | 98 | 3 | 1599 |
| 79 | OR498990 | Blood | 2018-03-15 | 1 | 12 | 3 | 2 | 2 | 98 | 3 | 1599 |
| 80 | OR498991 | Blood | 2018-03-17 | 1 | 12 | 3 | 2 | 2 | 98 | 3 | 1599 |
| 81 | OR498992 | Blood | 2018-03-20 | 1 | 3 | 3 | 2 | 2 | 94 | 3 | 191 |
| 82 | OR498993 | Blood | 2018-03-20 | 1 | 3 | 3 | 2 | 2 | 142 | 3 | 451 |
| 83 | OR498994 | Blood | 2018-03-21 | 41 | 66 | 23 | 2 | 92 | 119 | 3 | NT |
| 84 | OR498995 | Blood | 2018-04-12 | 1 | 3 | 3 | 2 | 2 | 107 | 3 | 784 |
| 85 | OR498996 | Blood | 2018-04-13 | 1 | 3 | 3 | 2 | 2 | 96 | 3 | 195 |
| 86 | OR498997 | Blood | 2018-04-13 | 1 | 3 | 3 | 2 | 2 | 94 | 3 | 191 |
| 87 | OR498998 | Blood | 2018-04-20 | 1 | 3 | 3 | 2 | 2 | 96 | 3 | 195 |
| 88 | OR498999 | Blood | 2018-05-03 | 1 | 3 | 3 | 2 | 2 | 94 | 3 | 191 |
| 89 | OR499000 | Blood | 2018-05-08 | 1 | 3 | 3 | 2 | 2 | 94 | 3 | 191 |
| 90 | OR499001 | Blood | 2018-05-18 | 1 | 3 | 3 | 2 | 2 | 94 | 3 | 191 |
| 91 | OR499002 | Blood | 2018-05-31 | 1 | 3 | 3 | 2 | 2 | 94 | 3 | 191 |
| 92 | OR499003 | Blood | 2018-06-05 | 10 | 53 | 4 | 11 | 4 | 98 | 5 | 491 |
| 93 | OR499004 | Blood | 2018-06-05 | 1 | 3 | 3 | 2 | 2 | 94 | 3 | 191 |
| 94 | OR499005 | Blood | 2018-06-25 | 1 | 3 | 3 | 2 | 2 | 106 | 3 | 369 |
| 95 | OR499006 | Blood | 2018-07-24 | 1 | 3 | 3 | 2 | 2 | 94 | 3 | 191 |
| 96 | OR499007 | Blood | 2018-07-26 | 1 | 3 | 3 | 77 | 2 | 97 | 3 | NT |
| 97 | OR499008 | Blood | 2018-07-26 | 1 | 3 | 3 | 2 | 2 | 106 | 3 | 369 |
| 98 | OR499009 | Blood | 2018-07-27 | 1 | 3 | 3 | 2 | 2 | 142 | 3 | 451 |
| 99 | OR499010 | Blood | 2018-07-30 | 1 | 3 | 3 | 2 | 2 | 106 | 3 | 369 |
| 100 | OR499011 | Blood | 2018-08-17 | 10 | 53 | 4 | 11 | 4 | 98 | 5 | 491 |
| 101 | OR499012 | Blood | 2018-08-28 | 1 | 3 | 3 | 2 | 2 | 106 | 3 | 369 |
| 102 | OR499013 | Blood | 2018-08-29 | 1 | 3 | 3 | 2 | 2 | 106 | 3 | 369 |
| 103 | OR499014 | Blood | 2018-09-04 | 10 | 53 | 4 | 4 | 4 | 98 | 5 | NT |
| 104 | OR499015 | Blood | 2018-09-06 | 10 | 53 | 4 | 4 | 4 | 98 | 5 | NT |
| 105 | OR499016 | Blood | 2018-09-08 | 10 | 53 | 4 | 4 | 4 | 98 | 5 | NT |
| 106 | OR499017 | Blood | 2019-01-03 | 1 | 3 | 3 | 2 | 2 | 142 | 3 | 451 |
| 107 | OR499018 | Blood | 2019-01-02 | 1 | 3 | 3 | 2 | 2 | 94 | 3 | 191 |
| 108 | OR499019 | Blood | 2019-01-09 | 1 | 3 | 3 | 2 | 2 | 142 | 3 | 451 |
| 109 | OR499020 | Blood | 2019-01-11 | 1 | 3 | 3 | 2 | 2 | 96 | 3 | 195 |
| 110 | OR499021 | Blood | 2019-01-15 | 1 | 3 | 3 | 2 | 2 | 107 | 3 | 784 |
| 111 | OR499022 | Blood | 2019-01-16 | 1 | 3 | 3 | 2 | 2 | 107 | 3 | 784 |
| 112 | OR499023 | Blood | 2019-01-21 | 1 | 3 | 3 | 2 | 2 | 96 | 3 | 195 |
| 113 | OR499024 | Blood | 2019-01-30 | 1 | 3 | 3 | 2 | 2 | 142 | 3 | 451 |
| 114 | OR499025 | Blood | 2019-02-06 | 1 | 3 | 3 | 2 | 2 | 96 | 3 | 195 |
| 115 | OR499026 | Blood | 2019-02-20 | 1 | 3 | 3 | 2 | 2 | 94 | 3 | 191 |
| 116 | OR499027 | Blood | 2019-02-26 | 1 | 12 | 3 | 2 | 2 | 103 | 3 | 469 |
| 117 | OR499028 | Blood | 2019-04-05 | 1 | 3 | 3 | 2 | 2 | 97 | 3 | 208 |
| 118 | OR499029 | Blood | 2019-04-10 | 1 | 3 | 3 | 2 | 2 | 94 | 3 | 191 |
| 119 | OR499030 | Blood | 2019-04-15 | 1 | 3 | 3 | 2 | 2 | 96 | 3 | 195 |
| 120 | OR499031 | Blood | 2019-04-16 | 1 | 3 | 3 | 2 | 2 | 96 | 3 | 195 |
| 121 | OR499032 | Blood | 2019-04-26 | 1 | 3 | 3 | 2 | 2 | 142 | 3 | 451 |
| 122 | OR499033 | Blood | 2019-05-04 | 1 | 3 | 3 | 2 | 2 | 94 | 3 | 191 |
| 123 | OR499034 | Blood | 2019-05-08 | 1 | 3 | 3 | 2 | 2 | 96 | 3 | 195 |
| 124 | OR499035 | Blood | 2019-12-27 | 1 | 3 | 3 | 2 | 2 | 97 | 3 | 208 |
| 125 | OR499036 | Blood | 2020-01-02 | 1 | 3 | 3 | 2 | 2 | 96 | 3 | 195 |
| 126 | OR499037 | Blood | 2020-06-11 | 1 | 3 | 3 | 2 | 2 | 96 | 3 | 195 |
| 127 | OR499050 | Blood | 2020-06-04 | 1 | 3 | 3 | 2 | 2 | 94 | 3 | 191 |
| 128 | OR499051 | Blood | 2020-06-13 | 1 | 12 | 3 | 2 | 2 | 145 | 3 | 357 |
| 129 | OR499052 | Blood | 2020-06-13 | 1 | 3 | 3 | 2 | 2 | 96 | 3 | 195 |
| 130 | OR499053 | Blood | 2020-06-17 | 1 | 3 | 3 | 2 | 2 | 96 | 3 | 195 |
| 131 | OR499054 | Blood | 2020-06-19 | 1 | 3 | 3 | 2 | 2 | 96 | 3 | 195 |
| 132 | OR499055 | Blood | 2020-06-25 | 1 | 3 | 3 | 2 | 2 | 107 | 3 | 784 |
| 133 | OR499056 | Blood | 2020-06-27 | 1 | 3 | 3 | 2 | 2 | 96 | 3 | 195 |
| 134 | OR499057 | Blood | 2020-07-18 | 1 | 3 | 3 | 2 | 2 | 94 | 3 | 191 |
| 135 | OR499058 | Blood | 2020-07-24 | 1 | 3 | 3 | 2 | 2 | 96 | 3 | 195 |
| 136 | OR499059 | Blood | 2020-08-04 | 1 | 3 | 3 | 2 | 2 | 107 | 3 | 784 |
| 137 | OR499060 | Blood | 2020-08-17 | 1 | 3 | 3 | 2 | 59 | 96 | 3 | 1653 |
| 138 | OR499061 | Blood | 2020-08-20 | 1 | 12 | 3 | 2 | 2 | 103 | 3 | 469 |
| 139 | OR499062 | Blood | 2020-08-28 | 1 | 3 | 3 | 2 | 2 | 107 | 3 | 784 |
| 140 | OR499063 | Blood | 2020-08-31 | 1 | 3 | 3 | 2 | 2 | 96 | 3 | 195 |
| 141 | OR499064 | Blood | 2020-09-07 | 1 | 3 | 3 | 2 | 2 | 142 | 3 | 451 |
| 142 | OR499065 | Blood | 2020-09-10 | 1 | 3 | 3 | 2 | 2 | 96 | 3 | 195 |
| 143 | OR499066 | Blood | 2020-10-02 | 1 | 3 | 3 | 2 | 2 | 107 | 3 | 784 |
| 144 | OR499067 | Blood | 2020-10-05 | 1 | 3 | 3 | 2 | 2 | 96 | 3 | 195 |
| 145 | OR499068 | Blood | 2020-10-10 | 1 | 3 | 3 | 2 | 2 | 142 | 3 | 451 |
| 146 | OR499069 | Blood | 2020-10-19 | 1 | 3 | 3 | 2 | 2 | 96 | 3 | 195 |
| 147 | OR499071 | Blood | 2020-10-20 | 10 | 53 | 4 | 11 | 4 | 98 | 5 | 491 |
| 148 | OR499070 | Blood | 2020-10-22 | 1 | 3 | 3 | 2 | 2 | 142 | 3 | 451 |
| 149 | OR499072 | Blood | 2020-10-29 | 1 | 3 | 3 | 2 | 2 | 94 | 3 | 191 |
| 150 | OR499073 | Blood | 2020-10-30 | 1 | 3 | 3 | 2 | 2 | 96 | 3 | 195 |
| 151 | OR499074 | Blood | 2020-11-02 | 1 | 3 | 3 | 2 | 2 | 96 | 3 | 195 |
| 152 | OR499075 | Blood | 2020-11-19 | 1 | 3 | 3 | 2 | 2 | 94 | 3 | 191 |
| 153 | OR499076 | Blood | 2020-11-24 | 1 | 3 | 3 | 2 | 2 | 94 | 3 | 191 |
| 154 | OR499077 | Blood | 2020-12-05 | 1 | 3 | 3 | 2 | 2 | 96 | 3 | 195 |
| 155 | OR499078 | Blood | 2020-12-08 | 1 | 3 | 3 | 2 | 2 | 142 | 3 | 451 |
| 156 | OR499079 | Blood | 2020-12-10 | 1 | 3 | 3 | 2 | 2 | 142 | 3 | 451 |
| 157 | OR499080 | Blood | 2020-12-17 | 1 | 3 | 3 | 2 | 2 | 94 | 3 | 191 |
| 158 | OR499081 | Blood | 2020-12-27 | 1 | 3 | 3 | 2 | 2 | 96 | 3 | 195 |
| 159 | OR499038 | Blood | 2021-02-03 | 1 | 3 | 3 | 2 | 2 | 96 | 3 | 195 |
| 160 | OR499039 | Blood | 2021-02-12 | 1 | 3 | 3 | 2 | 2 | 107 | 3 | 784 |
| 161 | OR499040 | Blood | 2021-02-15 | 1 | 3 | 3 | 2 | 2 | 96 | 3 | 195 |
| 162 | OR499041 | Blood | 2021-02-15 | 1 | 3 | 3 | 2 | 2 | 96 | 3 | 195 |
| 163 | OR499042 | Blood | 2021-02-19 | 1 | 3 | 3 | 2 | 2 | 96 | 3 | 195 |
| 164 | OR499043 | Blood | 2021-02-26 | 1 | 3 | 3 | 2 | 2 | 94 | 3 | 191 |
| 165 | OR499044 | Blood | 2021-04-08 | 1 | 3 | 3 | 2 | 2 | 94 | 3 | 191 |
| 166 | OR499045 | Blood | 2021-05-21 | 1 | 12 | 3 | 2 | 2 | 98 | 3 | 1599 |
| 167 | OR499046 | Blood | 2021-05-25 | 1 | 3 | 3 | 2 | 2 | 142 | 3 | 451 |
| 168 | OR499047 | Blood | 2021-06-03 | 1 | 3 | 3 | 2 | 2 | 94 | 3 | 191 |
| 169 | OR499048 | Blood | 2021-06-05 | 1 | 3 | 3 | 2 | 2 | 94 | 3 | 191 |
| 170 | OR499049 | Blood | 2021-07-31 | 1 | 3 | 3 | 2 | 2 | 106 | 3 | 369 |
